# COVID-19 Pandemic and Public Health Ethics in Japan

**DOI:** 10.31662/jmaj.2022-0119

**Published:** 2022-11-30

**Authors:** Eisuke Nakazawa

**Affiliations:** 1 Department of Biomedical Ethics, University of Tokyo, Tokyo, Japan

**Keywords:** public health ethics, medical ethics, COVID-19

## Abstract

Public health ethics is a discipline dealing with ethical issues relating to public health. As a branch of medical ethics, it also deals with clinical and research ethics. The core issue of public health ethics is to balance the conflict between individual liberty and the public good. Because of the coronavirus disease 2019 (COVID-19) pandemic, deliberation based on public health ethics is required to reduce social disparities and increase community cohesion. This study presents three public health ethics challenges. The first is to introduce an egalitarian liberal approach to public health concerning social and economic issues experienced by vulnerable populations both domestically and globally. I then propose alternative and compensatory public health policies that serve the principles of justice. Second, public health ethics must ensure procedural justice in all public health policy decisions. For example, when deciding to implement public health policies, including restrictions on individual liberties, the decision making process must be open to the public. Third, citizens and students must be educated on public health ethics. The public must be provided with an open forum to deliberate on ethical issues related to public health as well as the appropriate training to do so.

## Introduction

Public health ethics deals with ethical issues related to public health. As a branch of medical ethics, it also deals with clinical ethics and research ethics ^(1)^. Contemporary public health ethics is a relatively new area of inquiry that originated in the late 20th century. It became consolidated in the Journal of Public Health Ethics during the 2000s. Public health ethics is a field covering hygiene, epidemiology, sociology, ethics, philosophy of science, and political philosophy. As public health is policy oriented, the concepts of political philosophy form the core of public health ethics (Table 1).

**Table 1.** Representative Examples of Political Philosophy Theories.

Political Philosophy	Basic Value	Policy Features
Utilitarianism	Welfare	Consequentialist, maximum happiness of the greatest number
Liberal egalitarianism	Equality Reduction of disparities	Redistribution of goods
Libertarianism	Individual freedom Proprietary right	Small government
Communitarianism	Common good	Emphasis on community cohesion

**Figure 1.**
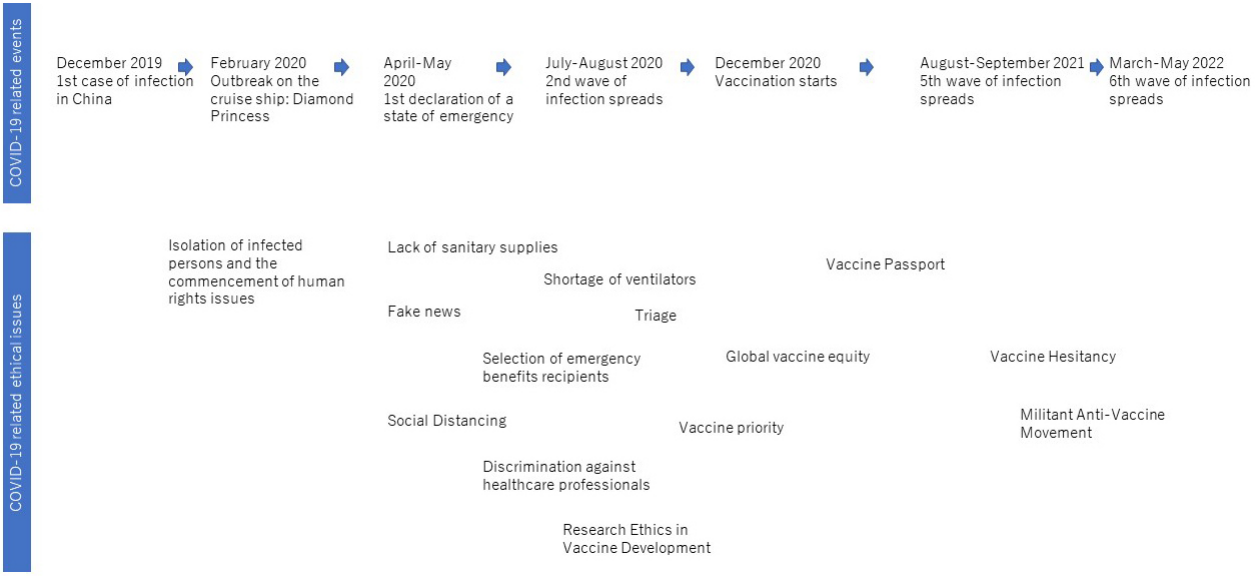
COVID-19-related events and ethical issues The upper panel shows COVID-19-related events (spikes in infections, policies such as the declaration of a state of emergency, and the beginning of vaccinations) by time. The lower panel shows ethical issues that arose in chronological order based on the timeline. The ethical issues listed are the author’s original selection.

The coronavirus disease 2019 (COVID-19) pandemic led to unprecedented interest in public health ethics in Japan. As Figure 1 shows, the 3-year pandemic period created public health ethical issues, such as the quarantine on the cruise ship *Diamond Princess*, business restrictions in restaurants, and vaccine hesitancy. These issues have been discussed in various forums such as national assemblies and government councils and through a variety of media and social networking sites.

This study examines the key questions behind these public health ethics issues and presents three challenges for Japanese society.

## Core Questions of Public Health Ethics

**Figure 2.**
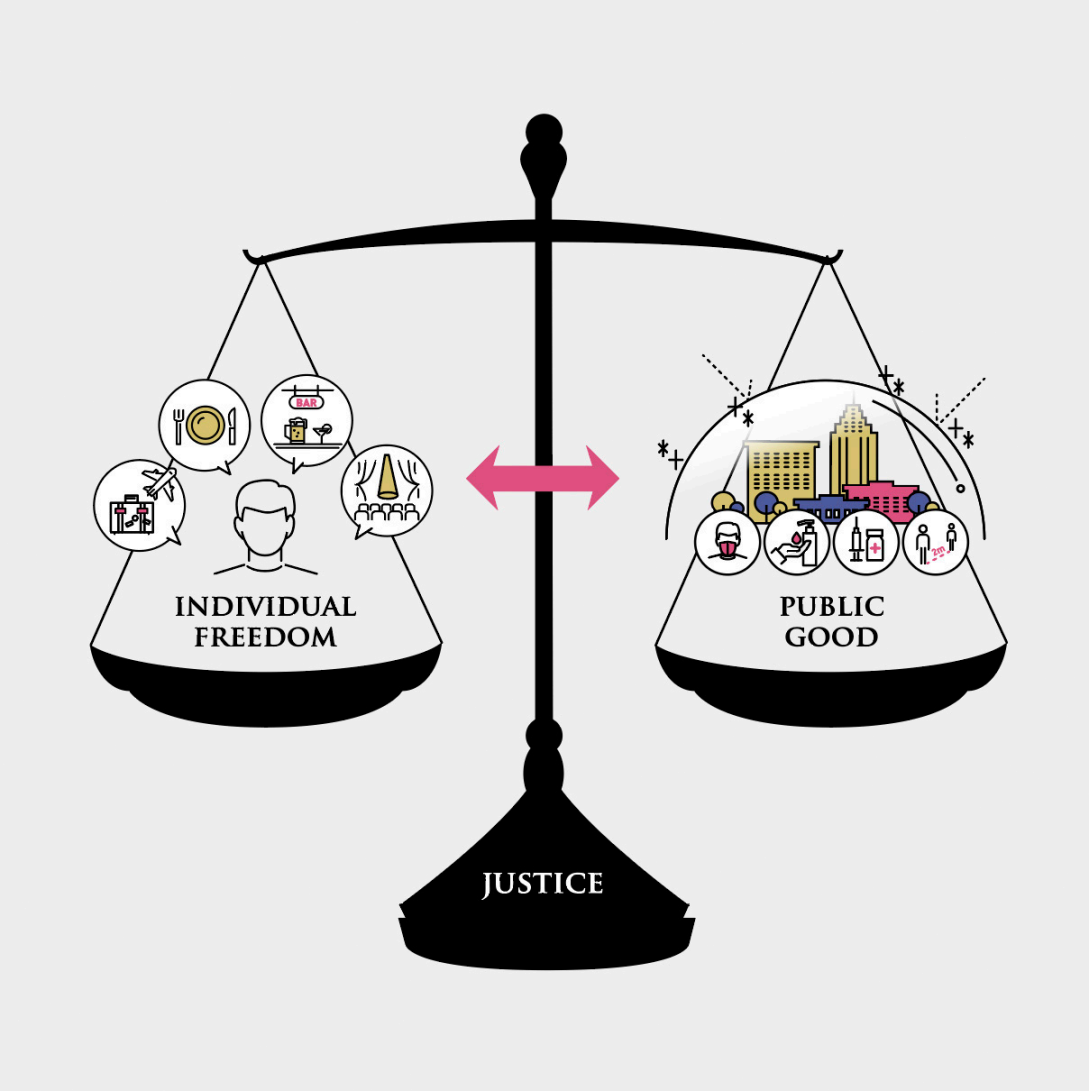
Basic structure of public health ethics The fundamental structure underlying many issues that arise in public health ethics involves the conflict between individual freedom and the public good. In line with the principle of justice, the central issue in public health ethics will be to adjust the balance between individual freedom and the public good. This is an original figure created by the author.

A basic requirement of a liberal society is to respect individual freedom as much as possible. Therefore, Japan’s pervasive liberalism is partly responsible for the Japanese government’s consistent caution with legally enforcing disease control measures against COVID-19. As the urban lockdowns implemented in many countries to prevent the spread of COVID-19 have shown, public health policies involve restrictions on individual freedom. The goal of lockdowns is to prevent the spread of infection and promote the good of society (public good). In this regard, the role of public health ethics is to provide (1) an ethical rationale for limiting individual freedom on account of public health interventions aimed at promoting the public good and (2) additional safeguards to reduce individual burdens and protect individual rights. The related policies must be consistent with the principles of natural justice and equality and consider the diverse core values ^(2)^. In sum, the core public health ethics problem is to reconcile the conflict between individual liberty and public value (Figure 2) ^(3)^.

How can Japanese society as a whole help in reconciling these value conflicts? Compared with the forced lockdowns implemented overseas, Japan’s COVID-19 policy placed relatively more emphasis on individuals’ autonomy ^(4)^. Although the voluntary soft lockdown policy may have been ethically desirable in that it respected individuals’ autonomy, it also blurred the placement of responsibility. This may have led to the outcome in which socially vulnerable populations, which would have been greatly influenced by the voluntary lockdown, received insufficient support. Indeed, economic and social burdens in Japan fall heavily on socially vulnerable populations, such as the elderly, women, and foreign residents. Reducing social disparities and increasing community cohesion are important values worth pursuing within the framework of public health ethics of the pandemic, within which the freedom of individuals conflicts sharply with public health values. COVID-19 is a disease of disparities ^(5)^. Against this backdrop, I present three challenges to public health ethics posed by the COVID-19 pandemic.

## Challenge 1: Advocacy for Vulnerable Populations

The first foundational challenge is to introduce an egalitarian liberal perspective into public health. Problems of inequality such as isolation of the elderly, poverty among young women, and employment constraints for foreigners under the foreign technical internship program have become commonplace during the COVID-19 pandemic and are increasing in severity ^(6)^. The situation of these vulnerable populations has been further weakened by social distancing.

When the discussion is deepened even further, it becomes clear that the elderly, women, and foreigners are not the only vulnerable populations. Vulnerable populations are quite diverse, and rather than presenting as a fixed social group, they vary by context. The COVID-19 pandemic has made us see certain groups being placed in vulnerable situations, who were not considered vulnerable prior to the pandemic. For instance, online lectures have isolated students. Furthermore, due to the cancellation of school programs, several students missed out on once-in-a-lifetime experiences. Finally, the discrimination encountered by health care personnel, who were ignored for no apparent reason during the pandemic, has become a social issue.

The COVID-19 pandemic, in combination with the public health policies that came into force to control infection, has widened these disparities. Notwithstanding the pandemic, these inequalities must be addressed in the interest of natural justice. The government has implemented several policies to reduce social and economic disparities. For example, they have provided benefits to low-income groups, but these have been inadequate to effect real change.

**Figure 3.**
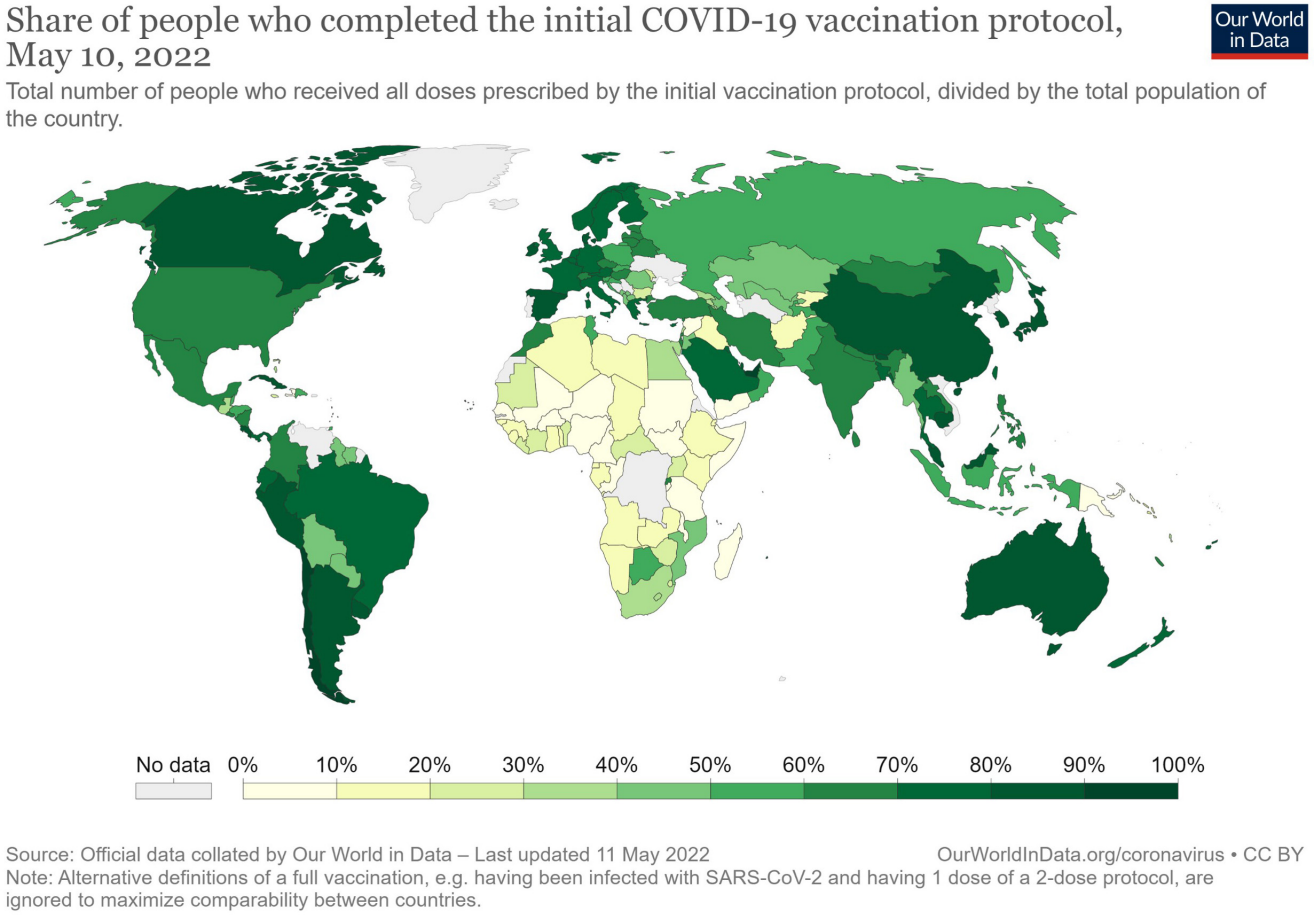
Global distribution of vaccine This world map shows the unequal global distribution of the vaccine as of May 10, 2022. The map clearly shows the delay in the provision of the vaccine to Central Asia, Western Asia, and African countries. This figure was created using data from Our World in Data (https://ourworldindata.org/).

While focusing on the correction of disparities and redistribution of goods with justice as the central principle (liberal egalitarianism), public health policies must also defend the rights of vulnerable populations and thereby reduce social disparities. Furthermore, the COVID-19 pandemic has shown that to overcome infectious diseases, we must first address international social and economic disparities. For example, as the global distribution of vaccines suggests (Figure 3), Japan and other wealthy countries also have obligations in the pursuit of global justice.

In summary, public health ethics should consider the social and economic problems of vulnerable populations, both domestically and globally, and formulate alternative and compensative public health that serves the principles of natural justice. Furthermore, if vulnerable populations are prone to change during socially abnormal situations such as a pandemic, then public health ethics must identify these socially vulnerable groups and adjust public health policies accordingly.

## Challenge 2: Procedural Justice

The second challenge is to ensure procedural justice in public health policy decisions such as restaurant restrictions or vaccine priorities, which affect the behavior of many people. Such public health policies must be implemented after careful deliberation, although these decisions should be taken quickly to control the spread of COVID-19 in a timely manner.

Particularly, the decision making process for implementation of public health policies, including restrictions on individual liberties, should be open to the public ^(7)^. An institutional design that increases the transparency of the process and allows for postevaluation is essential for public health policy decision making, and this must be addressed urgently as a matter of procedural justice.

## Challenge 3: Education and Civic Virtue

The third challenge is the education of the population at all levels. Citizens can support administrative efforts to increase transparency in policy decision making and ensure procedural justice through active political participation. Public health ethics education is essential to foster citizens’ political participation in policy decisions. Students must cultivate the ability to actively participate in politics at the school level and consider public health policies their own as future citizens. In their secondary and early postsecondary education, in particular, students should learn the fundamentals of public health ethics, including political philosophy and ethical theory based on practical examples. Participation in a variety of media, including social networking services, is also part of political life. Moreover, enhancing information literacy is a part of public health ethics education.

From the perspective of civic education, experts should provide appropriate information and create a forum for deliberation and discussion. Again, the characteristics of social media must be considered. Creating forums to pursue public health issues and the ethical issues associated with policies will help people develop civic virtues and encourage them to be actively involved in public health policy making.

## Conclusion

This study discusses the basic structure of public health ethics in Japan and some challenges, taking the COVID-19 pandemic as an example. To reconcile the conflict between individual freedom and public interest from the perspective of public health ethics, we must reduce the social disparities in Japan and enhance community cohesion. Furthermore, the study suggests the need to support vulnerable populations, procedural justice, and the cultivation of civic virtues through education. Moreover, health care professionals as well as the public should continuously explore the state of public health ethics in Japan.

## Article Information

### Conflicts of Interest

None

### Author Contributions

The author plays:

Substantial contributions to the conception or design of the work;

drafting the work or revising it critically for important intellectual content;

final approval of the version to be published; and

agreement to be accountable for all aspects of the work in ensuring that questions related to the accuracy or integrity of any part of the work are appropriately investigated and resolved.

### Approval by Institutional Review Board (IRB)

Not applicable

## References

[1] Akabayashi A, Kodama S. Nyumon Iryorinrigaku III: Koshueiseirinri [Introduction to medical ethics III: public health ethics]. Tokyo: Keiso Shobo; 2015. p. 11-24.

[2] Dawson A. What is ‘public health ethics?’ Akabayashi A ed. Oxford: Oxford University Press; 2014. The future of bioethics: international dialogues; p. 529-38.

[3] Nakazawa E, Ino H, Akabayashi A. Chronology of COVID-19 cases on the Diamond Princess cruise ship and ethical considerations: a report from Japan. Dis Med Public Health Preparedness. 2020;14(4):506-13. 10.1017/dmp.2020.50 PMC7156812 32207674

[4] Watanabe T, Yabu T. Japan’s voluntary lockdown. PLoS One. 2021;16(6):e0252468. 34111163 10.1371/journal.pone.0252468 PMC8191912

[5] Abrams EM, Szefler SJ. COVID-19 and the impact of social determinants of health. Lancet Respir Med. 2020;8(7):659-61. 32437646 10.1016/S2213-2600(20)30234-4 PMC7234789

[6] Nakazawa E, Akabayashi A. Pandemic Ethics: From COVID-19 to Disease X. Oxford (UK): Oxford University Press, in press; Chapter 15, Public health ethical and human rights considerations with physical distancing during pandemic.

[7] Daniels N. Accountability for reasonableness. BMJ. 2000;321(7272):1300-1. 11090498 10.1136/bmj.321.7272.1300 PMC1119050

